# Mitofusin 2 Dysfunction and Disease in Mice and Men

**DOI:** 10.3389/fphys.2020.00782

**Published:** 2020-07-09

**Authors:** Gerald W. Dorn

**Affiliations:** Center for Pharmacogenomics, Department of Internal Medicine, Washington University School of Medicine, St. Louis, MO, United States

**Keywords:** mitochondrial fusion, mitophagy, mitochondrial transport, Charcot-Marie-Tooth disease, neurodegeneration

## Abstract

A causal relationship between Mitofusin (MFN) 2 gene mutations and the hereditary axonal neuropathy Charcot-Marie-Tooth disease type 2A (CMT2A) was described over 15 years ago. During the intervening period much has been learned about MFN2 functioning in mitochondrial fusion, calcium signaling, and quality control, and the consequences of these MFN2 activities on cell metabolism, fitness, and development. Nevertheless, the challenge of defining the central underlying mechanism(s) linking mitochondrial abnormalities to progressive dying-back of peripheral arm and leg nerves in CMT2A is largely unmet. Here, a different perspective of why, in humans, MFN2 dysfunction preferentially impacts peripheral nerves is provided based on recent insights into its role in determining whether individual mitochondria will be fusion-competent and retained within the cell, or are fusion-impaired, sequestered, and eliminated by mitophagy. Evidence for and against a regulatory role of mitofusins in mitochondrial transport is reviewed, nagging questions defined, and implications on mitochondrial fusion, quality control, and neuronal degeneration discussed. Finally, in the context of recently described mitofusin activating peptides and small molecules, an overview is provided of potential therapeutic applications for pharmacological enhancement of mitochondrial fusion and motility in CMT2A and other neurodegenerative conditions.

## Overview of Mitofusins

Mitofusin (MFN) 1 and MFN2 are closely related dynamin family GTPases encoded by nuclear genes, but located on outer mitochondrial membranes. Originally described by Margaret Fuller and colleagues as the human homologs of Drosophila fuzzy onion (Fzo) (Santel and Fuller, 2001; Santel et al., 2003), MFN1 and MFN2 differ in catalytic GTPase activity essential for mitochondrial fusion (Ishihara et al., 2004), in their ability to localize at endo/sarcoplasmic reticulum and mediate trans-organelle calcium signaling (De Brito and Scorrano, 2008; Chan, 2012; Naon et al., 2016; Seidlmayer et al., 2019; Dorn, 2020), and in their proposed roles mediating mitophagic mitochondrial removal (Chen and Dorn, 2013; Gong et al., 2015). Nevertheless, MFN1 and MFN2 have largely overlapping functions in the sequential tethering and fusion of mitochondria and appear to be almost interchangeable in this regard. Thus, forced expression or pharmacological activation of either MFN1 or MFN2 can overcome mitochondrial fusion defects resulting from haplo-insufficiency in MFN1 or MFN2 null cells (Chen et al., 2003; Rocha et al., 2018), or from dominant suppression by MFN2 mutants (Detmer and Chan, 2007; Rocha et al., 2018; Zhou et al., 2019). Yet, detailed structures of individual mitofusin proteins located on outer mitochondrial membranes, of the oligomeric MFN structures that presumably mediate membrane deformation required for membrane fusion, and of trans MFN-MFN dimers that extend outward from mitochondria into cytosolic space and tether mitochondria together, are currently lacking. Consequently, much of the structural information about MFNs and the macromolecular complexes they form is either hypothetical or inferential (Koshiba et al., 2004; Brandt et al., 2016; Franco et al., 2016; Mattie et al., 2018; Rocha et al., 2018; Li et al., 2019).

By comparison, our phenomenological understanding of mitofusin pathophysiology is quite detailed, having been informed by gene knockout experiments (Chen et al., 2003, 2007, 2010, 2011, 2014; Lee et al., 2012; Sebastian et al., 2012, 2016; Kasahara et al., 2013; Song et al., 2014, 2015, 2017; Mourier et al., 2015; Boutant et al., 2017; Ramirez et al., 2017; Zhao et al., 2018; Bell et al., 2019; Han et al., 2020) and gain- or loss-of-function manipulation with MFN-specific mini-peptides and small molecules (Franco et al., 2016; Rocha et al., 2018). Collectively, this body of work supports three general conclusions: 1. Mitofusin insufficiency or impaired activity is broadly detrimental; 2. Absence of MFN2 is more deleterious than absence of MFN1; and 3. The consequences of impaired MFN-mediated mitochondrial fusion, mitophagy, and inter-organelle communication differ, depending upon cellular context.

Results of MFN deletion using *in vitro* cell and *in vivo* mouse models have built a solid foundation for interrogating the consequences of mitofusin deficiency produced by naturally occurring genetic *MFN* mutations in humans. Only two human diseases are unambiguously attributed to functional MFN deficiency: Charcot-Marie-Tooth disease type 2A (CMT2A) that is almost always mono-allelic (i.e., heterozygous) with autosomal dominant inheritance (*vide infra*), and multiple symmetric lipomatosis that has been described with bi-allelic (i.e., homozygous or compound-heterozygous) *MFN2* mutations (Rocha et al., 2017; Capel et al., 2018). Because very little is known about MFN-induced lipomatosis, here I focus on CMT2A.

## *MFN2* Mutations and Charcot-Marie-Tooth Disease

**FIGURE 1:**
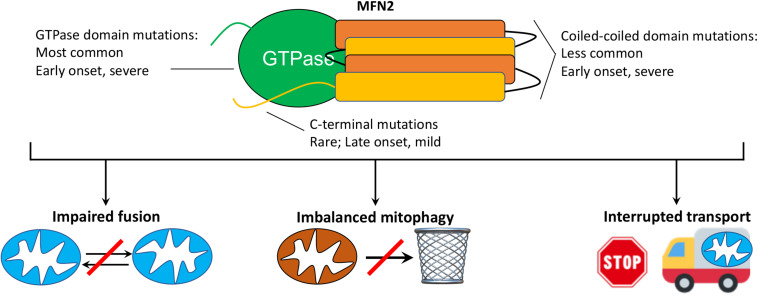
MFN2 mutation class and possible mechanisms mediating CMT2A. **(top)** Schematic depiction of MFN2 protein showing globular GTPase domain (green) and alpha helical coiled-coil stalk region (orange, yellow) and disease characteristics of respective mutations. CMT2A transgenic mice described herein express MFN2 R94Q and T195M, which are within the GTPase domain. **(bottom)** Possible cellular mechanisms by which dominant suppression of normal MFN1 and MFN2 function by damaging MFN2 mutations might provoke disease.

*Charcot-Marie-Tooth disease* was originally described in 1886 as “progressive muscular atrophy” by French neurologists Jean-Martin Charcot and Pierre Marie, and the English neurologist Howard Henry Tooth. As currently used in the clinic, the term CMT generally describes a large number of clinically heterogenous and genetically diverse inherited peripheral neuropathies (Fridman et al., 2015). CMT type 2A (CMT2A), defined by its causal *MFN2* gene mutations (Zuchner et al., 2004), is the second most common form of CMT (Cornett et al., 2016) and is remarkable for early onset and rapid progression during childhood. In the largest reported United States study (*n* = 99 patients from Wayne State University plus 27 patients from the United Kingdom) (Feely et al., 2011), the average age of onset for CMT2A was 4.4 years (vs 41 years for other forms of CMT2); most CMT2A patients were non-ambulatory by 20 years of age. These findings are consistent with the International Neuropathies Consortium cohort of >1,400 CMT patients (including *n* = 910 CMT1 and 237 CMT2) (Fridman et al., 2015). *MFN2* mutations comprise ∼6% of familial CMT (∼22% of familial CMT2) (Bombelli et al., 2014; Choi et al., 2015) and are, depending upon nationality, the second or third most common cause of CMT, after the *PMP22* duplication/deletion and *GJB1* mutations that cause CMT1A (Fridman et al., 2015; Ando et al., 2017; Hoebeke et al., 2018; Yoshimura et al., 2019). Among patients with CMT2A, the majority of *MFN2* mutations affect the amino terminal GTPase and mid-protein MFN2 coiled-coiled domains, with disease onset in the first 2 years of life and an aggressive clinical course. A few patients have mutations at the extreme MFN2 carboxyl terminus and typically exhibit later onset (range 5–33 years old) and milder disease (Feely et al., 2011; Stuppia et al., 2015; Figure 1). Strikingly, CMT provoked by vincristine therapy has been described in children treated for acute lymphoblastic leukemia (Chauvenet et al., 2003), providing an example of gene-environment interactions that can induce the clinical phenotype.

The prototypical CMT2A patient exhibits loss of sensory and motor nerve function in the distal limbs (Bombelli et al., 2014), but some patients additionally show evidence of laryngeal paralysis (Zuchner et al., 2006), loss of visual acuity from retinal degeneration (Chung et al., 2006; Verhoeven et al., 2006; Zuchner et al., 2006; Feely et al., 2011), sensorineural hearing loss (Nicholson et al., 2008), spinal cord atrophy (Bombelli et al., 2014), and upper motor neuron signs (Vucic et al., 2003; Chung et al., 2006; Rance et al., 2012), which support reports of central nervous system involvement (Brockmann et al., 2008; Boaretto et al., 2010; Milley et al., 2018). Proximal limb weakness, in addition to classical distal limb involvement, is observed in approximately one third of CMT2A patients (Bombelli et al., 2014), and occurs earlier than in CMT1A. Clinical heterogeneity observed in CMT2A has been attributed in part to the different functional impact of multiple implicated *MFN2* mutations: GTPase and coiled-coiled domain mutations tend to induce more severe and early onset disease whereas mutations in the carboxy terminal domain tend to have later onset and milder disease (Feely et al., 2011; Stuppia et al., 2015). However, even within family members having the same MFN2 mutation there can be inter-individual variability in disease manifestation and progression (Chung et al., 2006; Bombelli et al., 2014).

An unequivocal diagnosis of CMT2A requires genetic testing that identifies a causal *MFN2* mutation in a suggestive clinical context. Disease severity and progress can be monitored by tests of neuromuscular integrity that reveal areflexia (from lower sensory-motor nerve damage), loss of vibratory and proprioceptive senses (Gemignani et al., 2004; Park et al., 2012), muscle weakness and atrophy (especially in the tibialis anterior muscle, resulting in foot-drop; Neves and Kok, 2011); neuroelectrophysiological testing shows normal nerve conduction velocity with reduced compound motor activation potentials (CMAP) (Harding and Thomas, 1980; Berciano et al., 2017). Histologically, there is loss of large myelinated fibers with partial regeneration, but normal myelin (Muglia et al., 2001; Verhoeven et al., 2006); neuronal and skeletal muscle mitochondria are abnormal on ultrastructural examination (Verhoeven et al., 2006; Sole et al., 2009).

Although the genetic cause of CMT2A is, by definition, mutational MFN2 dysfunction (Zuchner et al., 2004; Bombelli et al., 2014), the functional consequences linking MFN2 dysfunction to the cellular pathology underlying neuronal die-back and neuromuscular degeneration remain unclear (reviewed in Filadi et al., 2018). Mechanisms that have been proposed include: 1. Disrupted mitochondrial fusion and loss of fusion-related homeostatic repair (Chen and Chan, 2006; Pareyson et al., 2015); 2. Related interruption of mitochondrial-endoplasmic reticular communications that variably perturb calcium crosstalk and phospholipid/cholesterol synthesis (Larrea et al., 2019); 3. Dysregulated mitochondrial quality control with mitochondrial depletion or retention of cytotoxic senescent and damaged mitochondria (Rizzo et al., 2016; Filadi et al., 2018); and 4. Interrupted mitochondrial trafficking through neuronal axons (Baloh et al., 2007; Pareyson et al., 2015; Crunkhorn, 2018; Figure 1). While it seems likely that all of these mechanisms contribute in a combinatorial way to CMT2A neuromuscular disease, disrupted mitochondrial trafficking is particularly intriguing as a contributory mechanism because a transportation defect could explain why CMT2A preferentially affects the longest peripheral nerves innervating lower and upper limbs; travel delays have greater impact on long journeys.

## *In vivo* Pathophysiology Provoked by Human CMT2A MFN2 Mutants Expressed in Mice

Two CMT2A MFN2 mutants, MFN2R94Q and MFN2T105M, have been expressed in mice by various groups attempting to evoke features of peripheral axonopathy similar to the human condition. Both of these mutations are in the MFN2 GTPase domain, and expression of either dominantly inhibits mitochondrial fusion and induces mitochondrial clustering in fibroblasts (Detmer and Chan, 2007). It is notable that CMT2A MFN2 mutants induce phenotypes that recapitulate some features of human CMT2A (detailed below), whereas expressing wild-type (WT) MFN2 in the same experimental systems is well tolerated. Absence of pathology provoked by overexpressed WT MFN2 in mice suggests greater cell capacity to compensate for mitofusin gain of function than for mitofusin inhibition or gene deletion.

**FIGURE 2:**
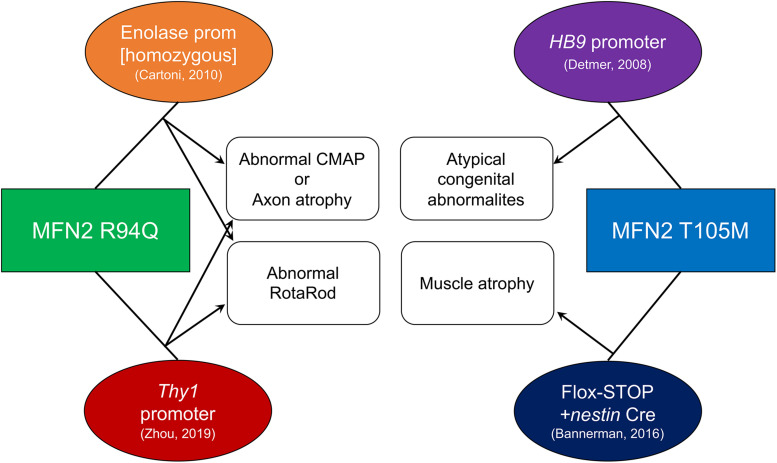
Technical and phenotypic characteristics of published CMT2A mouse models. Mutant MFN2 transgenes (squares) were expressed using systems shown in ovals. Resulting phenotypes are connected with arrows. References for each mouse are in parentheses.

The first reported CMT2A MFN2 mutant mouse was developed in David Chan’s laboratory and used a motor-neuron specific HB9 promoter to drive expression of a bi-cistronic MFN2T105M/EGFP transgene in motor neurons (Detmer et al., 2008; Figure 2). Because H9B gene activity is highest during fetal development (Arber et al., 1999; Thaler et al., 1999), HB9-MFN2T105M transgene expression in these mice decreased after birth and was not detected from P10 (Detmer et al., 2008). Thus, these mice reflect developmental motor neuron phenotypes that are not reversible by perinatal extinction of pathological MFN2 mutant expression. Nevertheless, histological loss of motor neuron axons and mitochondrial dysmorphology with uneven distribution throughout axons were associated in homozygous transgenic mice with abnormal musculoskeletal hindlimb development and loss of tibialis anterior muscle mass. These developmental mouse phenotypes are not typical for human CMT2A in which the disease first manifests in early childhood. Additionally, early transgene extinction does not recapitulate the human genetic condition. Thus, the utility of these mice, which are no longer available, was limited.

A ROSA-targeted flox-stop transgene approach to MFN2T105M expression was more recently employed by David Pleasure to work around potential transgene insertion effects and avoid perinatal extinction of transgenes being driven by developmentally important gene promoters (Bannerman et al., 2016; Figure 2). A flox-stop MFN2 T105M transgene was knocked into the genetically safe mouse Rosa26 locus where its transcription is driven by the powerful hybrid CAG (CMV enhancer fused to chicken beta-actin) promoter. A loxP-flanked STOP cassette makes the MFN2 T105M transgene silent until Cre-recombination. Thus, tissue-specific MFN2T105M expression can be accomplished by combining the flox-stop transgene with a tissue-specific Cre. Crossing the MFN2T105M mice with nestin-Cre (expressed in neuroectoderm) provoked gait abnormalities and hind-limb muscle (i.e., soleus and tibialis anterior) muscle fiber atrophy with evidence for diminished mitochondria in nerve axons. Surprisingly, in the 10 weeks old male mice described, axon size and RotaRod latency (time before falling off a rotating cylinder, which measures neuromuscular functional integrity) were normal. The ROSA-STOP-(CAG-MFN2T105M) mice are available from The Jackson Laboratory (stock number 025322) and have also been used in combination with motor neuron-specific HB9-Cre to elicit mitochondrial hypomotility in sciatic nerve axons studied *ex vivo* (Rocha et al., 2018); an *in vivo* phenotype of the latter mice has not been reported.

MFN2R94Q was first expressed in mice by Chrast and Martinou using the neuron-specific enolase promoter, designated “MitoCharc” mice (Cartoni et al., 2010; Figure 2). Heterozygous (MitoCharc1) and homozygous (MitoCharc2) mice exhibited age-dependent (5 months) decreases in RotaRod latency with gait abnormalities, associated at 1 year with axons having smaller diameters and increased numbers mitochondria. In a follow-up study, diminished mitochondrial fusion, impaired mitochondrial-ER communication with induction of an ER stress response, and defective mitochondrial transport were all implicated in observed neuromuscular dysfunction. The heterozygous MitoCharc1 mice are available from The Jackson Laboratory (stock number 012812).

More recently, Robert Baloh transgenically expressed MFN2R94Q in mice using a *Thy1* promoter that expresses in most neuronal cell types (Zhou et al., 2019; Figure 2). Compared to other CMT2A mouse models, this one exhibits a constellation of abnormalities that closely resembles neuromuscular pathology in human CMT2A. In functional studies, compared to both normal controls and WT MFN2 transgenic mice, MFN2 R94Q mice exhibited fewer rearing events on open field testing, decreased RotaRod latency, and diminished grip strength, all reflecting impaired motor function. Additionally, MFN2R94Q mice showed poor visual acuity by optokinetic testing, recapitulating retinal (sensory neuron) involvement sometimes seen in human CMT2A (Zuchner et al., 2006). Histological studies showed axon degeneration and atrophy with mitochondrial fragmentation and aggregation in the absence of mitophagy. Importantly, forced concomitant expression of WT MFN1 with MFN2R94Q prevented the CMT2A phenotypes, suggesting a role for the putative imbalance between MFN1 and MFN2 in CMT2A mitochondrial and neuromuscular defects. However, the notion that correcting an MFN isoform imbalance (and not simply restoring overall MFN expression/activity to more normal levels) evoked phenotypic rescue in this mouse requires that MFN2 expression fail to provide the same benefits as MFN1 expression. Although the MFN2 rescue mice were described, only a comparable rescue of body weight was reported; functional neuromuscular phenotyping of mice expressing WT MFN1 and MFN2R94Q is needed to address questions of MFN subtype effects. All three mouse strains in this study are available from The Jackson Laboratory (stock numbers 029745, 032728, and 033391).

Phenotypic heterogeneity in these CMT2A mice might be the consequence either of the different transgenic systems used to express mutant MFN2 (at different levels and in different neuronal subtypes) or to the different MFN2 mutants examined. Since the two versions of MFN2R94Q mice have different phenotypes, it is the author’s opinion that transgene system, rather than mutation identity, is likely the greatest source of experimental variability. An obvious work-around that can control for different transgene expression systems is to knock human CMT2A mutations into analogous positions within the mouse genome. Indeed, the knock-in approach has been used to insert MFN2R94Q into the mouse genome (Strickland et al., 2014). Heterozygous MFN2R94Q knock-in mice (i.e., that recapitulate the mono-allelic human condition) showed decreased open field movement, but other read-outs of motor dysfunction like RotaRod latency, grip strength, and beam traversing, were normal. Also there was no evidence for sensory neuron dysfunction in tests of thermal allodynia. Likewise, axon diameter and mitochondrial movement in dorsal root ganglion neurons were normal. By comparison, MFN2R94Q mice bred to homozygosity died from unidentified reasons within 24 h of birth. Thus, these MFN2 mutant knock-in mice have not recapitulated seminal characteristics of human CMT2A. The reasons why transgenic overexpression of MFN2R94Q evokes CMT2A-like phenotypes in mice whereas a knock-in of the same mutation failed to do so are not entirely clear. Since expression level is a critical determinant of dysfunction provoked by dominant inhibitory proteins, it is possible that mutant transgenes were simply expressed at higher levels than the knock-in mutant alleles.

Charcot-Marie-Tooth disease type 2A is typically a disease of long peripheral nerves. In the classical presentation CMT2A affected children do not manifest signs until the “toddler” stage or somewhat later. The disease then progresses during childhood and adolescence and stabilizes in young adulthood (Feely et al., 2011; Fridman et al., 2015). If one views these data as a function of patient size rather than age (which are obviously highly correlated during childhood), the disease is silent in smaller humans, progresses as they enlarge, and then stops progressing when growth stops. Viewed in the context of characteristic involvement of long nerves, disease progression as nerves elongate supports a physical dimension to disease manifestation. If this is correct, then mice may be resistant to knock-in attempts to provoke the human disease because even their longest nerves (sciatic and tail) are short in comparison with human peripheral nerves. MFN2 mutant knock-ins of larger animal models (rats, dogs, pigs, primates) would therefore be expected to more closely reproduce clinical CMT2A.

## MFN-Mediated Fusion, Mitophagy, and Transport in Disease

As introduced above, it is widely accepted that mitochondrial fusion is essential to cell health. MFN1 and MFN2 are necessary for mitochondrial fusion, both as the initiators of enabling mitochondria-mitochondria tethering and as the mediators of outer membrane fusion. For these reasons, it is widely assumed that CMT2A, the prototypical disease of MFN dysfunction, is a disease of impaired mitochondrial fusion (Chan, 2012). Since MFN1 can generally substitute for MFN2 as a mitochondrial fusion factor (Detmer and Chan, 2007; Zhou et al., 2019), but only MFN2 mutations cause disease, it is worth exploring how impairment of other MFN2 functions might also contribute to pathological phenotypes.

The underlying rationale for defective mitochondrial fusion causing CMT2A is obvious: Mitochondrial fusion appears essential to the health of all tissues and cell types, independent of their mitochondrial density, morphology, or motility. Accordingly, complete abrogation of mitochondrial fusion by combined ablation of both MFN1 and MFN2 was detrimental to neurons that contain small, highly motile mitochondria (Chen et al., 2007), to cardiac and skeletal muscle that contain small, but stationary mitochondria (Chen et al., 2010, 2011; Song et al., 2015), as well as to fibroblasts whose mitochondria are elongated and interconnected into structurally pliable networks (Chen et al., 2003).

*Mitochondrial fusion* is an important mechanism by which mitochondrial content exchange maintains biochemical and genetic uniformity within the organelle collective. This process of “complementation” moderates the number and impact of mutations in the small mitochondrial genome (mtDNA) (Chan, 2012), which encodes 13 critical electron transport chain complex proteins essential to normal mitochondrial respiratory function (Chen et al., 2007, 2010, 2011). Moreover, the dynamic imbalance resulting from reduced mitofusin activity or expression, thus favoring mitochondrial fission over fusion, can lower the threshold for apoptotic programmed cell death (Frank et al., 2001; Sugioka et al., 2004). Given the apparent ubiquity and importance of mitochondrial fusion demonstrated *in vitro* and in multiple genetic mouse models, it seems paradoxical that in human patients the damaging effects of CMT2A-linked MFN2 mutations are restricted to distal neurons of the longest peripheral nerves. If the pathophysiology relates exclusively to impaired fusion, then one would predict that short and long nerves, and central and peripheral nerves, and other tissues with high mitochondrial density and metabolic demand (like cardiac and skeletal muscle), would all be affected. And if CMT2A is only caused by defective mitochondrial fusion, then severely damaging MFN1 mutations should also cause a CMT2A-like syndrome. It has been suggested that differences between MFN1 and MFN2 expression account for exclusivity of MFN2 mutations as causal factors in disease. Thus, if MFN2 is expressed in neuronal cells at levels far greater than MFN1, this could explain the unique role of MFN2 (Lee et al., 2012; Zhou et al., 2019). However, this notion has been difficult to validate at the protein level because of varying antibody affinities for the two mitofusins. Moreover, if increased MFN2/MFN1 expression is a specific feature of the neurons affected by CMT2A, it is hard to understand how this imbalance provokes CMT2A-induced neuronal damage that increases with distance from the spine, i.e., why within the same neuron the distal axon dies back, but the proximal axon and soma are spared. In this context it seems likely that other MFN2-mediated events, in addition to altered fusion, contribute to CMT2A.

*Mitophagy*, which shares some of the same protein mediators and can be co-regulated with apoptosis, can be considered as a form of “programmed mitochondrial death” (Whelan et al., 2012; Dorn and Kitsis, 2015). MFN2 plays a unique role in mitophagy compared to MFN1, and mitophagic dysfunction may also contribute to neuronal degeneration in CMT2A.

The classic mitophagy pathway is a mechanism for selective removal of irreparably damaged mitochondria from cells via the PINK1-Parkin pathway, as recently reviewed in detail (Dorn, 2016; Pickles et al., 2018). An *in vivo* role played by MFN2 as a signaling intermediate between mitochondrial PINK1 kinase and the cytosolic E3 ubiquitin ligase Parkin was described in neurons and cardiac myocytes (Lee et al., 2012; Chen and Dorn, 2013; Chen et al., 2014; Song et al., 2015, 2017). Briefly, nuclear-encoded PINK1kinase is translated in the cytosol and actively imported into mitochondria; in normal mitochondria PINK1 is immediately degraded via targeted proteolysis. Thus, healthy mitochondria contain little or no PINK1 protein. However, PINK1 degradation is suppressed in functionally impaired or damaged mitochondria, permitting PINK1 to accumulate. When present, PINK1 phosphorylates its many mitochondrial substrates, including at least three amino acids on MFN2 (Chen and Dorn, 2013; Rocha et al., 2018). MFN2 phosphorylation by PINK1 promotes MFN2-Parkin binding on the outer mitochondrial membrane, facilitating Parkin-mediated ubiquitination of one hundred or more mitochondrial outer membrane proteins (Sarraf et al., 2013). Ubiquitinated mitochondrial outer membrane proteins attract autophagosomes and stimulate autophagosomal engulfment of the damaged organelle, thus initiating mitophagy (Pickles et al., 2018).

PINK1-phosphorylated MFN2 is not only a “Parkin receptor,” but it loses the ability to promote mitochondrial fusion (Chen and Dorn, 2013; Rocha et al., 2018). This binary functional switching of MFN2 from mitochondrial fusion protein to mitophagy effector serves to sequester organelles destined for mitophagic elimination from fusing with healthy members of the mitochondrial collective, thus preventing “mitochondrial contagion” (Bhandari et al., 2014). In this context, it is reasonable to postulate that fusion-impaired MFN2 mutants could also be mitophagy-impaired. However, impaired mitophagy is not a feature of CMT2A. Indeed, both an *in vitro* study of iPSC-derived motor neurons derived from siblings harboring a MFN2A383V mutation (Rizzo et al., 2016), and an *in vivo* mouse study of MFN2R94Q (Zhou et al., 2019), suggest that mitophagy is either not changed or is increased in experimental CMT2A, perhaps as a compensatory reaction to respiratory dysfunction in fusion-defective organelles.

*Mitochondrial transport* requires organelle coupling via the ortholog of *Drosophila* Milton, Trak1, to dynein and kinesin, the molecular motors that move cargo along cell microtubular structures (Stowers et al., 2002). Because the Trak1-dynein/kinesin-microtubule pathway is a general mechanism for trans-cellular cargo transport, for mitochondria to be transported as the cargo a mitochondrial Trak1 binding protein Mitochondrial Rho GTPase 1 (Miro1) attaches and detaches mitochondria from the cell transport apparatus (Wang and Schwarz, 2009). By binding to Trak1, Miro1 protein located (along with MFN1 and MFN2) on outer mitochondrial membranes can attach the mitochondria to molecular motors of the transport apparatus, and release the mitochondria where cytosolic calcium levels are locally increased (Devine et al., 2016). Consistent with this mechanism, Miro-deficient cells have impaired mitochondrial transport (Lopez-Domenech et al., 2018).

Published data have implicated MFN proteins in mitochondrial transport, which could explain selective die-back of long peripheral nerves in CMT2A. However, a plausible molecular mechanism by which mitofusins engage the transport machinery has not been defined, and the relative roles of putative MFN- vs canonical Miro-driven mitochondrial trafficking are unclear. Evidence for diminished mitochondrial motility in neurons expressing CMT2A MFN2 mutants is inferential (Baloh et al., 2007; Misko et al., 2010; Rocha et al., 2018) and potentially explainable by indirect effects of impaired mitochondrial fusion (and resulting alterations in mitochondrial morphology, connectivity, and respiratory function) on mitochondrial motility. The observation that both mitofusins can interact with Miro proteins when the protein pairs are co-expressed (Misko et al., 2010) suggested direct interactions between MFN1 or MFN2 and the canonical Miro-Trak mitochondrial transport apparatus. Again however, because co-expressed proteins can participate in non-physiological promiscuous interactions, and co-immunoprecipitation may involve third unidentified protein partners, these types of co-IP studies do not prove either a naturally occurring or direct interaction between MFNs and Miros. Thus, while published associations between mutational or allelic loss-of-MFN function and mitochondrial dysmotility suggest that mitofusins somehow either regulate or help mediate mitochondrial transport, this notion requires unambiguous delineation of the causal molecular events in the context of endogenous proteins in relevant neuronal systems.

## Pharmacological Mitofusin Activation as a Research Tool and Potential Clinical Therapeutic

Until recently the only way to interrogate mitofusin function was genetically, through gene deletion or overexpression of WT or mutant MFNs. Several years ago, Franco et al. described an MFN2-derived activating mini-peptide that, by competing with endogenous peptide-peptide interactions constraining MFN1 and MFN2 into closed “inactive” conformations, induced a more open and “active” conformation capable of promoting mitochondrial fusion (Franco et al., 2016). The MFN activator peptide is useful as a research tool for *in vitro* studies of MFN functioning (Zhou et al., 2019), but peptides are unwieldy as clinical therapeutics because they are expensive to produce, usually cannot be administered orally, and tend to be rapidly eliminated. Thus, Rocha et al. used rational design to develop small molecule peptidomimetics with even greater *in vitro* potency than the original mitofusin activating peptide: First, a minimally effective MFN activating peptide of 11 amino acids was identified. Then, alanine scanning was used to delineate the functionally important amino acids. Finally, a pharmacophore model was used to perform an *in silico* screen for commercially available compounds having similar structural characteristics as the function-critical agonist peptide amino acids. Biological screening of 55 such candidates identified two with detectable agonist activity and *de novo* synthesis of so-called “Franken-molecules” having different chemical moieties from these fusogenic compounds led to a first-in-class small molecule mitofusin agonist, Chimera B-A/l (Rocha et al., 2018). Chimera is a potent mitochondrial fusogen (EC50 3–5 nM) lacking activity for related dynamin-family proteins, which rapidly (<1 h) normalized mitochondrial transport in *ex vivo* sciatic nerve axons from CMT2A mice (Rocha et al., 2018). Because the pharmacokinetic and *in vivo* pharmacodynamic characteristics of Chimera B-A/l were not reported it is not clear whether this compound will prove useful *in vivo* as a treatment for CMT2A. Nevertheless, the possibility of using pharmacology, instead of or in addition to genetic manipulation, to understand the various roles played by mitofusins in cell biology and neurodegenerative disease adds to the existing research tool kit and may hold clinical promise.

## Author Contributions

GD wrote the manuscript and created the figures.

## Conflict of Interest

GD is an inventor on patent applications PCT/US17/052556 submitted by Stanford University and Washington University, PCT/US18/028514 submitted by Washington University, and PCT/US19/46356 and PCT/US20/14784 submitted by Mitochondria Emotion, Inc., that cover the use of peptides or small molecule mitofusin agonists to treat chronic neurodegenerative diseases. GD is the founder of Mitochondria in Motion, Inc., a Saint Louis based biotech R&D company focused on enhancing mitochondrial trafficking and fitness in neurodegenerative diseases.
